# Cerebral amyloid angiopathy-related inflammation: a case report presenting with a rare variant in SORL1 gene

**DOI:** 10.1186/s12883-019-1326-2

**Published:** 2019-05-15

**Authors:** Yanjiao Du, Chao Liu, Congmin Ma, Xiaohui Xu, Xufeng Zhou, Haitao Zhou, Chao Huang

**Affiliations:** 1 Department of Neurology, Luoyang Central Hospital Affiliated to Zhengzhou University, NO. 288, Middle Zhongzhou Road, Xigong Square, Luoyang, 471000 China; 2 Department of Radiology, Luoyang Central Hospital Affiliated to Zhengzhou University, NO. 288, Middle Zhongzhou Road, Xigong Square, Luoyang, 471000 China

**Keywords:** CAA-ri, ApoE, SORL1, Corticosteroid, Recurrence

## Abstract

**Background:**

Cerebral amyloid angiopathy-related inflammation (CAA-ri) is a rare clinical entity, characterized by headaches, seizures, rapidly progressive cognitive decline, behavioral changes and magnetic resonance imaging (MRI) findings underlying the autoimmune and inflammatory reaction at the level of CAA-affected vessel. CAA-ri is likely responsive to corticosteroid. MRI shows asymmetric and multifocal white matter hyperintensity (WMH) lesions and multiple cerebral microbleeds. Apolipoprotein E (ApoE) ε4 homozygosity is associated with CAA-ri strongly [Neurology 68(17):1411-1416, 2007, Ann Neurol 73(4):449-458, 2013, J Alzheimers Dis 44(4):1069-1074, 2015]. SORL1 processes a causal involvement in Alzheimer’s disease (AD) as a proposed modulator of the amyloid precursor protein (APP). It is unclear whether SORL1 is involved with CAA-ri or not.

**Case presentation:**

A 48-year-old woman suffered from a one-day history of a headache, nausea, and vomiting. Neurological examination revealed normal. We diagnosed this case as probable CAA-ri according to the clinic manifestations and MRI. Gene detection indicated a rare variant in SORL1 and ApoE ε4 homozygosity. When treated with corticosteroid, the patient’s clinical symptoms and MRI manifestations were almost relieved. However, when keeping the corticosteroid withdrawal for three months, the patient relapsed with a headache and typical images on MRI emerged. Corticosteroid therapy was effective again. Unfortunately, susceptibility weighted imaging (SWI) showed increased microbleeds. With tapering corticosteroid slowly, no recurrence was found on this patient with four-month follow-up.

**Conclusion:**

A variant of SORL1 may be associated with CAA-ri, recurrence of disease could be detected with MRI by an increased microbleeds. Our case report suggests that corticosteroid therapy might be effective for CAA-ri.

## Background

Cerebral amyloid angiopathy (CAA) is presented with progressive deposition of amyloid proteins within the cortical and leptomeningeal arteries, which is a common pathology in the elder [1, 2]. In recent years, studies show that coexisting inflammations found in CAA patients, such as vasculitis or perivasculitis, have been recognized as CAA-related inflammation (CAA-ri) [3]. CAA-ri is a rare clinical entity, characterized by headaches, seizures, rapidly progressive cognitive decline, behavioral changes and MRI findings underlying the autoimmune and inflammatory reaction at the level of CAA-affected vessel [4–7]. CAA-ri is thought to result from an inflammatory response to β-amyloid (Aβ) protein in the blood vessel walls [5] and likely responsive to corticosteroid or other immunosuppressive agents [7]. According to the pathology, CAA-ri can be divided into two subtypes. Inflammatory CAA is perivascular infiltration around the vessel wall, and Aβ-related angiitis is characterized by transmural and intramural inflammation, often with gulomas formation [8, 9]. Amyloid-related imaging abnormalities (ARIA) represent the major severe side effect of Aβ immunotherapy for Alzheimer’s disease (AD), and characterize the acute phase of CAA-ri [9]. Hence, this supports a hypothesis that an immunological response to vascular amyloid, with increased vascular permeability, may be a common underlying pathophysiological mechanism of CAA-ri [10]. Apolipoprotein E (ApoE) ε4 homozygosity known to associate with increased burden of Aβ deposition in vessels [11], is strongly associated with CAA-ri [4, 5, 12]. In this case, we present a case of spontaneous CAA-ri with a variant in SORL1, which emerges as a promising candidate in control of the brain amyloidogenic processes in AD pathology [13] and with ApoE ε4 homozygosity.

## Case presentation

**Fig. 1:**
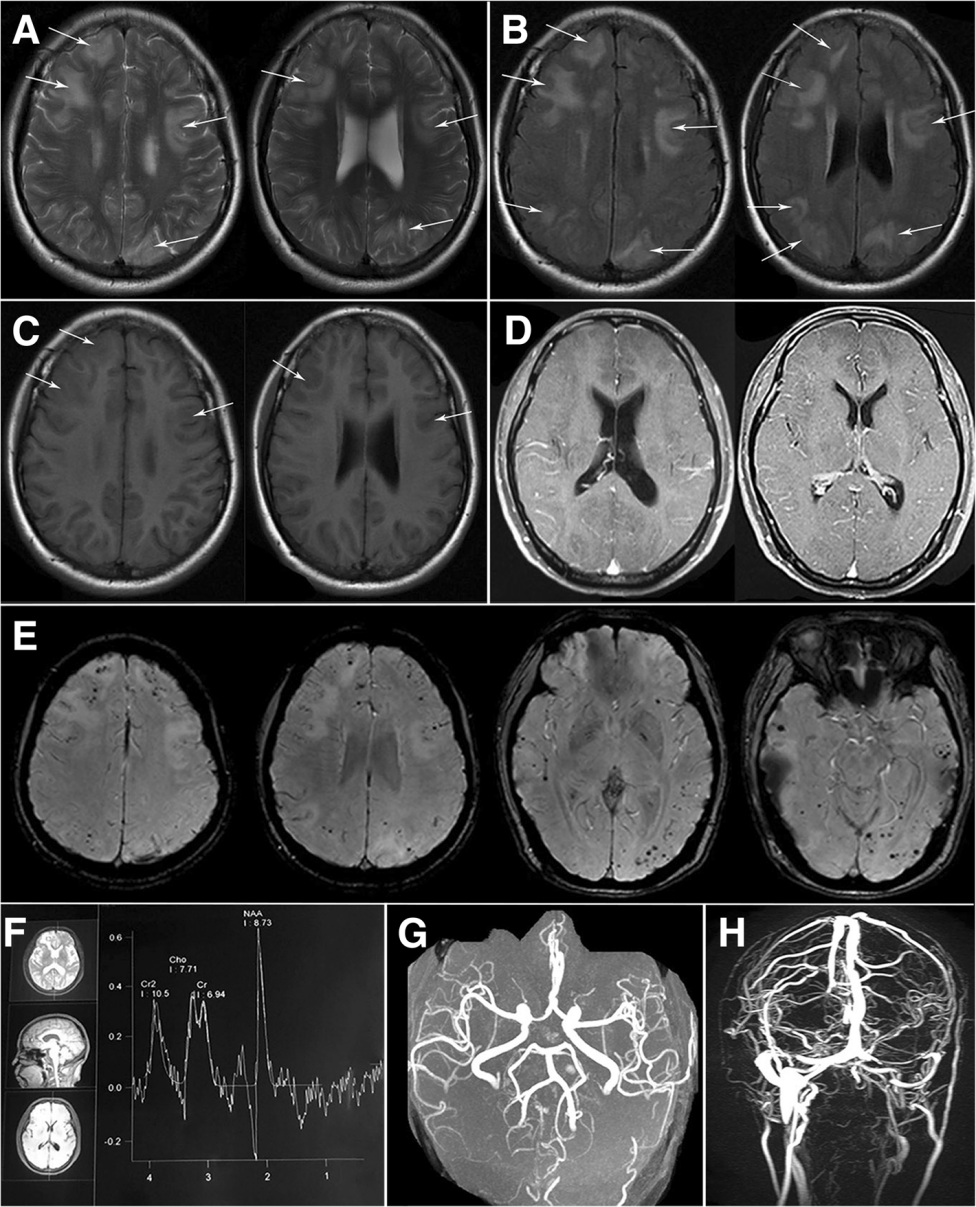
Brain magnetic resonance imaging (MRI) findings of our patient at the onset, **a**-**c** multifocal white matter hyperintensity lesions were revealed on T2 and fluid attenuated inversion recovery (FLAIR) and slight hypointense on T1 (arrows); **d** gadopentetate enhanced MRI showing no lesion was enhanced; **e**: susceptibility weighted imaging (SWI) showing multiple cerebral microbleeds in cortical and subcortical areas; **f** magnetic resonance spectrum demonstrating a normal spectrum in lesions; **g** magnetic resonance angiography was normal; **h**: magnetic resonance venography showing thinness in the bilateral transverse and left sigmoid sinus and decreased signal intensity

**Fig. 2:**
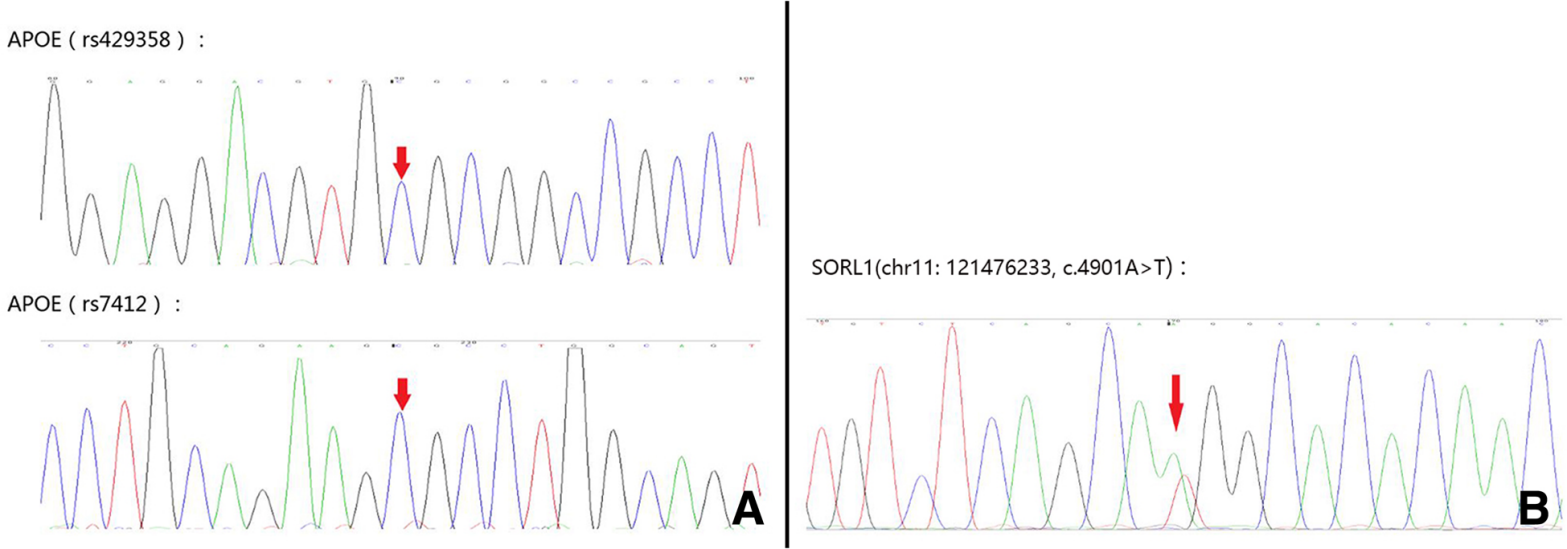
Gene detection results of the patient. **a** Apolipoprotein E ε4 homozygosity, **b** a variant in SORL1:c.4901A>T

A 48-year-old woman presented to our outpatient neurology with a headache, nausea, and vomiting for one day. Her headache was persistent, tolerable and blunt in the right temporal region. She had a hypertension history for three years and had been controlled within normal range by antihypertensive drugs. She had no psychosocial and familial hereditary history. Neurological examination was normal. Routine blood tests, serum C-reactive protein level and erythrocyte sedimentation rate showed normally. Serum thyroid hormone and sex hormone level were also in the normal range. Serum anti-MPO and P-ANCA were weakly positive, while other indicators including antinuclear, anti-SSA, anti-SSB, anti-dsDNA, anti-Sm, anti-RNP, anti-Scl-70, anti-RP3, anti-GBM, anti-neutrophil cytoplasmic antibodies in cytoplasmic, antibodies to neuro-paraneoplastic syndromes, neuromyelitis optica (NMO), myelin basic protein (MBP) and myelin oligodendrocyte glycoprotein (MOG) in blood as well as cerebrospinal fluid (CSF) were negative. Serum tumor markers were negative. Lumber puncture showed higher intracranial pressure with 240 mmH_2_O. Subsequent CSF analysis showed normal protein, glucose, and cell count. Testing on the bacterial, viral, fungal and cryptococcal pathogens of CSF was negative. Magnetic resonance imaging (MRI) of the brain revealed multifocal white matter hyperintensity (WMH) lesions (cortico-subcortical) on T2 and fluid attenuated inversion recovery (FLAIR) associated mass effect, which were slight hypointense on T1 and didn’t enhance on gadopentetate enhanced MRI (Fig. 1a-d). Susceptibility weighted imaging (SWI) revealed multiple cerebral microbleeds in cortical and subcortical areas (Fig. 1e). Magnetic resonance spectrum (MRS) demonstrated a normal spectrum. (Fig. 1f). Magnetic resonance angiography showed normally (Fig. 1g). Magnetic resonance venogram showed thinness in the bilateral transverse and left sigmoid sinus and decreased signal intensity (Fig. 1h). Further digital subtraction angiography excluded venous sinus thrombosis. The genetic sequencing showed ApoE ε4 homozygosity and a variant in SORL1 (Fig. 2a-b).

**Fig. 3:**
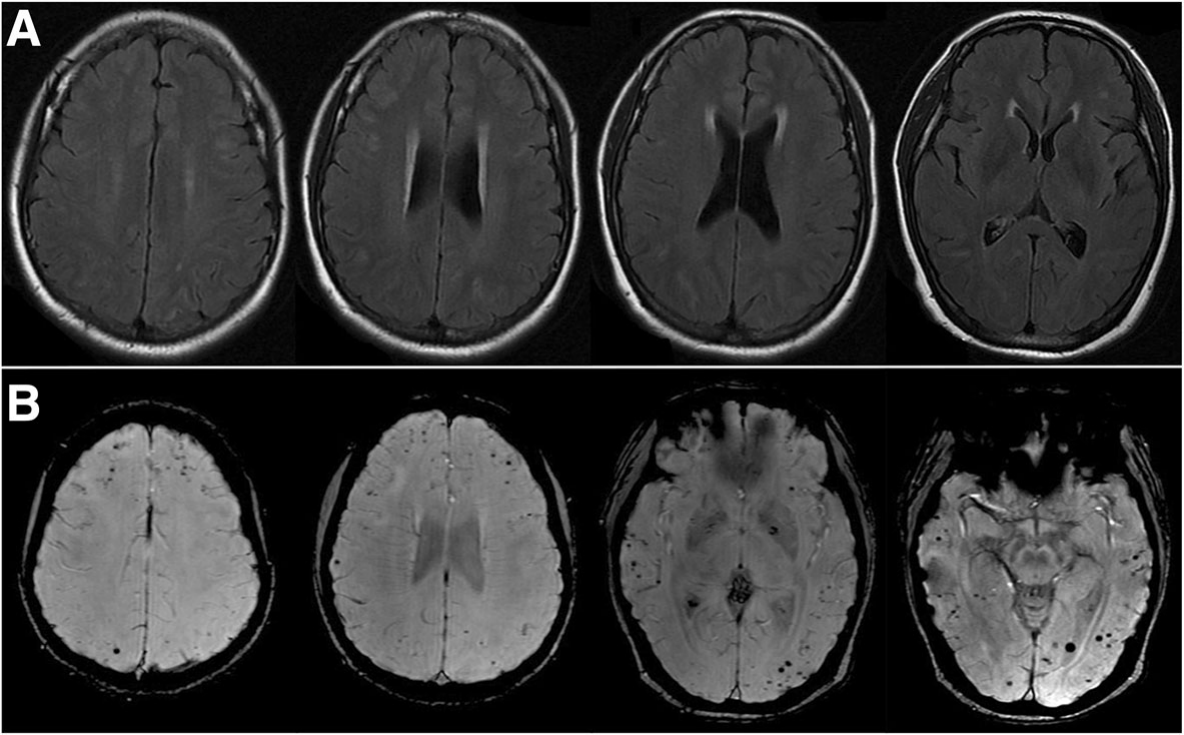
MRI findings after the first corticosteroid therapy. **a** FLAIR images showing lesions were almost completely disappeared; **b** SWI images showing multiple cerebral microbleeds remained unchanged

**Fig. 4:**
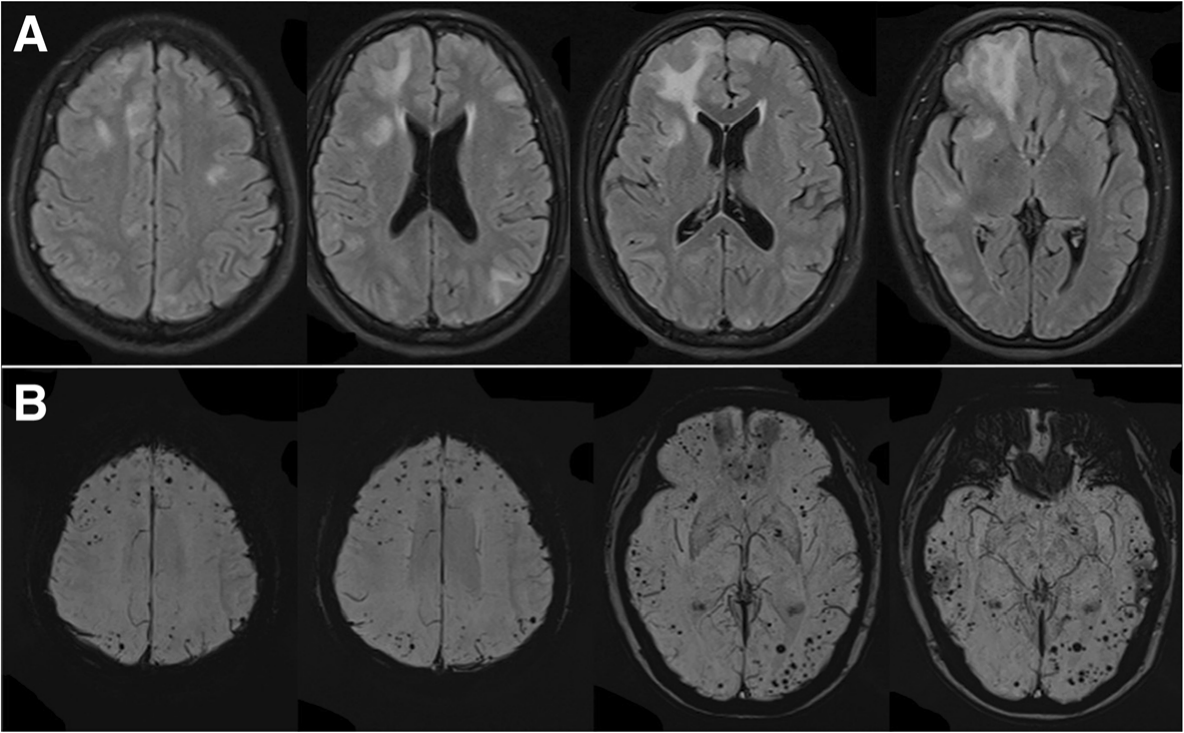
Recurrence MRI images after corticosteroid withdrawal for three months. **a** multifocal white matter hyperintensity lesions were revealed on FLAIR; **b** multiple cerebral microbleeds in cortical and subcortical areas increased on SWI

**Fig. 5:**
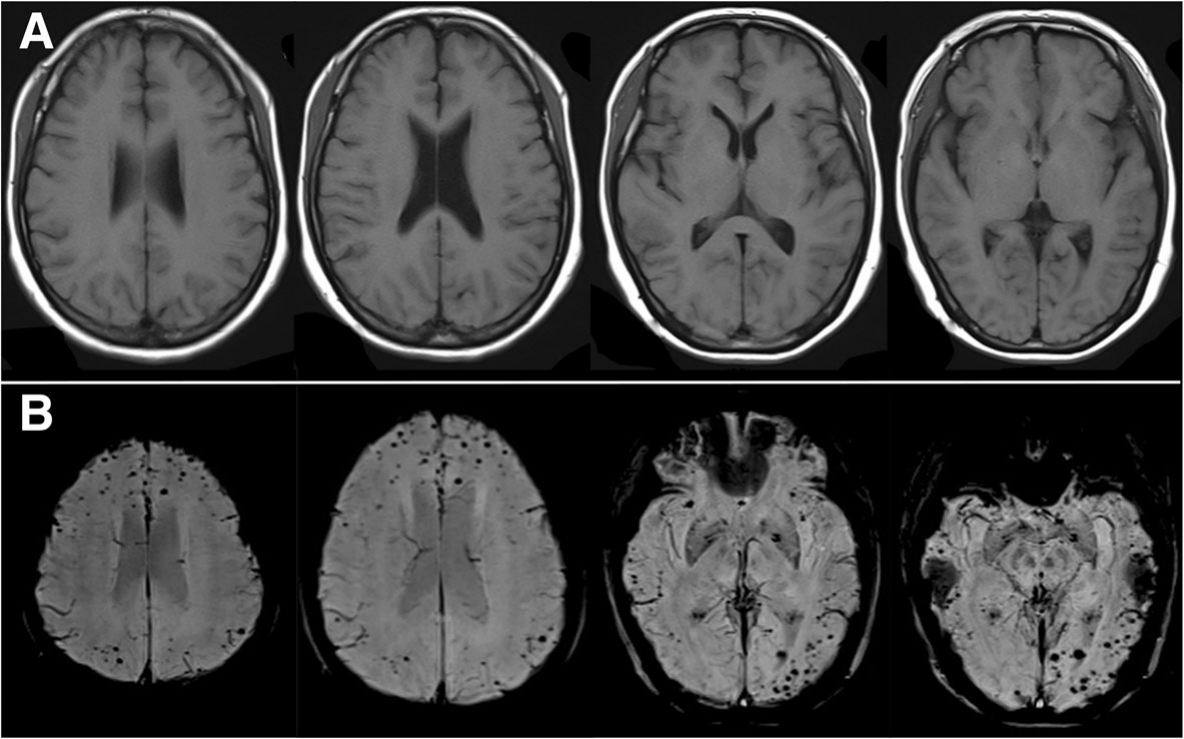
MRI images after the second corticosteroid therapy for four months. **a** no lesion was found on FLAIR; **b** microbleeds were not increased on SWI

Based on these findings, we made a diagnosis of probable CAA-ri. We firstly treated the patient with dexamethasone 10 mg/day via venous for three days and then reduced it by half for three days, followed by oral prednisolone 60 mg/day tapered within six weeks (a weekly reduction of 10 mg). On the 3rd day of treatment, her headache was significantly relieved. MRI demonstrated that the lesions on FLAIR almost completely disappeared six weeks later (Fig. 3a-b). She presented to our clinic with complaints of a headache again after corticosteroid withdrawal for three months. MRI indicated CAA-ri relapsed and SWI showed that the microbleeds in cortical/subcortical areas were increased (Fig. 4a-b). We launched the methylprednisolone 1000 mg pulse therapy for her. Lesions on T2/FLAIR disappeared quickly. We recommended reducing the dose of corticosteroid gradually until a long-term administration of oral prednisone 5 mg/day. No recurrence was found and the lesions of microbleeds were not increased any more over the next four-month follow-up (Fig. 5a-b).

## Discussion and conclusions

Definitive diagnosis of CAA-ri generally requires typical clinical manifestation, typical MRI features, and brain biopsy [8]. A study demonstrated image-guided stereotactic brain biopsy is usually safe, but complications may occur, with transient and permanent morbidity in 9 and 13% of cases [14]. Considering the risk of brain biopsy, Auriel, et al. validated the modified probable CAA-ri criteria, which is sufficiently specific to be incorporated into the clinical practice for identifying patients with good sensitivity and excellent specificity [15]. According to the criteria [15], we made a diagnosis of probable CAA-ri based on five aspects: (1) the patient’s age was over 40 years old; (2) the patient presented with headache but no acute intracerebral hemorrhage (ICH) was found; (3) MRI showed WMH lesions (cortico-subcortical) which were asymmetric without past ICH; (4) SWI revealed multiple cerebral microbleeds in cortical and subcortical areas; (5) No neoplastic, infectious or other indications were found according to the laboratory and imaging results. Lesions on T2/FLAIR revealed obvious edema and no lesion was enhanced in MRI gadolinium enhancement, which was similar to low-grade glioma and lymphoma [16]. However, they were excluded by a further MRS examination. The images of posterior reversible encephalopathy syndrome (PRES) was similar to CAA-ri. But PRES is always accompanied by hypertension combined with severe underlying diseases, including high blood pressure, pregnancy eclampsia, severe kidney disease, malignant tumor, systemic lupus erythematosus, etc. Due to the patient’s controlled normal blood pressure and none of the diseases mentioned above, we excluded PRES. The diagnosis of CAA-ri is considered as definite if, in addition to these criteria, a histopathologic examination indicates perivascular, transmural, or intramural inflammation and amyloid deposition within vessels of the affected area in the cortex and the leptomeninges [7]. However, our patient refused to take a brain biopsy.

The best therapy of CAA-ri remains to be determined. The presented data support to use the empirical immunosuppressive therapy (and avoiding brain biopsy) for patients who meet the criteria proposed for probable CAA-ri [7, 15]. A reasonable subsequent approach would be considered a brain biopsy in empirically treated patients who fail to respond to the corticosteroid therapy within three weeks [7]. Strict blood pressure control was also managed for patients with hypertension [17]. The primary treatment for our patient was dexamethasone 10 mg per day via venous infusion. Clinical symptoms and image almost completely relieved (Fig. 3). After keeping corticosteroid withdrawal for three months, the patient developed recurrence. She was treated with high-dose methylprednisolone, clinical symptoms and lesions on T2/FLAIR were completely relieved, which supported the diagnosis of CAA-ri [4, 6, 7]. When recurred, SWI showed increased microbleeds in the cortical/subcortical areas (Fig. 4). In the following four months, the lesions of microbleeds have not been increased, indicating that the microbleeds may be the result of the acute vascular inflammation of CAA-ri. To improve prognosis, large samples studies to further determine a better administration for CAA-ri are necessary.

Genetic detection of the patient indicated ApoE ε4 homozygosity and a variant in SORL1 (chromosome11, c.4901A>T) (Fig. 2), which was fairly rare in the normal population. Strong support for a causal involvement in neurodegenerative disease came with genetic studies associating SORL1 gene variants with the occurrence of sporadic AD [13]. SORL1 encodes a 250-kDa protein named sorting protein-related receptor with A-type repeats (SorLA), decreasing production and deposition of Aβ peptide as a proposed modulator of amyloid precursor protein (APP) processing [13, 18]. Aβ levels are mainly determined by the kinetic of SorLA and APP interaction according to the mathematic model which supports the fact that APP sorting pathway is a very important factor in defining the risk of AD [13, 19, 20]. CAA-ri is thought to result from an inflammatory response to Aβ protein in the blood vessel walls [5]. Spontaneous ARIA and CAA-ri have also been described in familial forms of AD (FAD), i.e., in β-amyloid protein precursor -amyloid protein precursor (AβPP) duplication carriers [21], in presenilin 1-associated FAD (I202F PSEN1 mutation) [12], and in two siblings carrying the P284S PSEN1 mutation [22]. Thus, SORL1 may be associated with CAA-ri. A variant in SORL1 may give rise to the risk of CAA-ri via immune response to Aβ excessive deposition. ApoE ε4 homozygosity known to associate with increased burden of Aβ deposition in vessels [11] is strongly associated with CAA-ri [4, 5, 12] and may potentiate the intense inflammatory response [23]. Investigation of the degree of CAA-ri and inflammation in SORL1 mutation with ApoE ε4 homozygosity would be an interesting avenue for future research.

A variant of SORL1 may be associated with CAA-ri, recurrence of disease could be detected with MRI by an increased microbleeds. Our case report suggests that corticosteroid therapy might be effective for CAA-ri.

## Abbreviations

AD: Alzheimer’s disease
ApoE: apolipoprotein E
APP: amyloid precursor protein.
ARIA: Amyloid-related imaging abnormalities
Aβ: β-amyloid
AβPP: β-amyloid protein precursor
CAA: cerebral amyloid angiopathy,
CAA-ri: cerebral amyloid angiopathy-related inflammation
CSF: cerebrospinal fluid
FLAIR: fluid attenuated inversion recovery
ICH: intracerebral hemorrhage
MBP: myelin basic protein
MOG: myelin oligodendrocyte glycoprotein
MRI: magnetic resonance imaging
MRS: magnetic resonance spectrum
NMO: neuromyelitis optica
PRES: posterior reversible encephalopathy syndrome
SorLA: sorting protein-related receptor with A-type repeats
SWI: susceptibility weighted imaging
WMH: white matter hyperintensity
